# Accuracy of pre-operative fistula diagnostics in anorectal malformations

**DOI:** 10.1186/s12887-021-02761-6

**Published:** 2021-06-16

**Authors:** Louise Tofft, Martin Salö, Einar Arnbjörnsson, Pernilla Stenström

**Affiliations:** 1 Department of Pediatric Surgery, Skåne University Hospital, Lasarettsgatan 48, S-221 85, Lund, Sweden; 2 Department of Clinical Sciences, Pediatrics, Lund University, Lasarettsgatan 48, S-221 85, Lund, Sweden

**Keywords:** Anorectal malformations, Fistula, Voiding cystourethrogram, Colostogram, Cystoscopy, Endoscopy

## Abstract

**Background:**

Surgical safety during posterior sagittal anorectal plasty (PSARP) for anorectal malformations (ARM) depends on accurate pre-operative fistula localization. This study aimed to evaluate accuracy of pre-operative fistula diagnostics.

**Methods:**

Ethical approval was obtained. Diagnostic accuracy of pre-PSARP symptoms (stool in urine, urine in passive ostomy, urinary tract infection) and examination modalities (voiding cystourethrogram (VCUG), high-pressure colostogram, cystoscopy and ostomy endoscopy) were compared to final intra-operative ARM-type classification in all male neonates born with ARM without a perineal fistula treated at a tertiary pediatric surgery center during 2001–2020.

**Results:**

The 38 included neonates underwent reconstruction surgery through PSARP with diverted ostomy. Thirty-one (82%) had a recto-urinary tract fistula and seven (18%) no fistula. Ostomy endoscopy yielded the highest diagnostic accuracy for fistula presence (22 correctly classified/24 examined cases; 92%), and pre-operative symptoms the lowest (21/38; 55%). For pre-operative fistula level determination, cystoscopy yielded the highest diagnostic accuracy (14/20; 70%), followed by colostogram (23/35; 66%), and VCUG (21/36; 58%). No modality proved to be statistically superior to any other.

**Conclusions:**

Ostomy endoscopy has the highest diagnostic accuracy for fistula presence, and cystoscopy and high-pressure colostogram for fistula level determination. Correct pre-operative ARM-typing reached a maximum of 60–70%.

## Background

Boys born with anorectal malformations (ARM) without a perineal fistula are suspected to have a recto-urinary tract fistula until proven otherwise [1]. These patients are commonly given a neonatal diverted ostomy [2, 3]. According to the Krickenbeck classification of ARM, recto-urinary tract fistulas are sub-divided into recto-bulbar, recto-prostatic, and recto-bladder neck fistulas [4]. A minority of patients present with no fistula [1].

The Krickenbeck classification not only predicts long-term outcome in ARM [5–7], but is also used for planning reconstructive surgery in detail. Accurate surgical work up prior to posterior sagittal anorectal plasty (PSARP) [8] is essential in order to plan surgery correctly and thereby increase surgical safety, minimize any risk of unnecessary surgical trauma or injuries to the urinary and genital tract, avoid the presence of remnants of fistulas, and to make an accurate decision as to whether or not to operate laparoscopically [9–12].

A standard method to estimate fistula presence is pre-operative registration of symptoms: stool-colored urine, urinary tract infection (UTI), and urine in diverted ostomy. Pre-operative radiologic examinations for fistula level determination traditionally include high-pressure colostogram [13], possibly combined with voiding cystourethrogram (VCUG) including additional urinary tract anomaly diagnostics [14, 15]. Other methods to establish uro-genital and fistula anatomy include peri-operative cystoscopy and ostomy endoscopy of the atretic rectum [16].

The diagnostic accuracy of high-pressure colostogram and VCUG vary from 52 to 100% according to the few previous published studies with fistula diagnostic accuracy data [10, 17, 18]. A pre-operative fistula diagnostic accuracy of 100% is unlikely according to our clinical experience. Establishing pre-operative fistula diagnostic accuracy of conventional modalities compared to definite ARM-subtyping during PSARP is not only important for patient surgical safety but is also essential for further development and assessment of upcoming modalities, such as high-frequency ultrasound and high-Tesla magnetic resonance imaging (MRI) [17–21].

The aim of this study was therefore to evaluate the diagnostic accuracy of pre-operative clinical symptoms, VCUG, high-pressure colostogram, and endoscopy of the urinary tract and diverted ostomy, regarding presence and location of fistulae compared to peri-operative findings in male neonates born with ARM.

## Methods

### Study design

This was a retrospective study of medical records of all male neonates born with ARM without a perineal fistula, treated at a tertiary center of pediatric surgery between January 2001 and October 2020. In 2018 the center was appointed as one of two national ARM-centers, thereby evolving from a low- to a high-volume center, now serving 5 million inhabitants. Patients’ medical records were reviewed regarding pre-operative diagnostic observations and examinations of fistula presence and location. All patients underwent surgical reconstruction according to the original PSARP-method [8] and they had annual follow-ups according to the local and national ARM-care programs.

### Inclusion and exclusion criteria

All male neonates born with ARM without a perineal fistula, treated with diverted ostomy and submitted to surgical work-up including fistula diagnostics prior to PSARP at the center, were included. Exclusion criteria were primary PSARP without diverted ostomy and PSARP performed elsewhere.

## Methods

Medical charts were reviewed regarding pre-operative clinical observations of stool-colored urine, urine in ostomy and UTI, X-ray reports of pre-operative VCUGs and high-pressure colostograms, and peri-operative examination findings of cystoscopies and endoscopies of diverted ostomies. Final ARM-type classifications during PSARP were noted.

### Diagnostics

Radiologic- and endoscopic examinations were conducted and the presence and location of a fistula was noted. VCUGs and high-pressure colostograms were performed according to standard clinical practice [14, 22] by five pediatric radiology specialists at an accredited radiology department. Colostograms were performed by a dynamic X-ray examination with a water-soluble contrast injection through a catheter with an inflated cuff balloon at the orifice of the passive stoma, creating intra-bowel pressure and a convex appearance of the atretic rectum, enabling fistula visibility. VCUGs were performed collecting evidence of vesico-urethral reflux, and by retracting the catheter slowly in the urethra under dynamic X-ray examination, enabling fistula visibility. Cystoscopies and endoscopies of diverted ostomies including fistula catheterization with a guide wire from the atretic rectum to the urinary tract [16] were performed during PSARP anesthesia by five pediatric surgeons or pediatric urologists.

### Statistical analysis

Descriptive data analyses and group comparisons were performed using Excel (Microsoft® Excel for Mac, version 16.16.8, 2018) and SPSS® (IBM® SPSS® Statistics, version 26, 2019). In group comparisons for dichotomous data, Fisher’s exact test was used while Mann–Whitney U-test was used for continuous parameters. Continuous data were not normally distributed and were therefore presented as median (min–max), and categoric data as absolute numbers and percentages, n (%).

Contingency tables of true outcome (final ARM-type classification during PSARP) and findings of symptoms and examination modalities were devised. Diagnostic accuracy (%) of each symptom and examination modality regarding ability of correct differentiation between fistula presence or absence was calculated by the proportion of true positive and true negative cases in all evaluated cases. Diagnostic accuracy (%) of examination modalities regarding ability of correct fistula level determination was calculated by the proportion of true positive and true negative cases in all evaluated cases. To compare the diagnostic ability regarding fistula presence or absence among symptoms and examination modalities, a receiver-operating characteristic (ROC) curve analysis was also used, with calculation of the area under the curve (AUC) and its 95% confidence interval (95%CI). A *p*-value of < 0.05 was considered significant.

### Ethics

This study was approved by the Regional Ethics Committee, Southern Region, Sweden (DNR 2017/191).

## Results

### Patients

Forty male neonates born with ARM without perineal fistulas were identified in the hospital records. One was excluded due to primary PSARP without diverted ostomy and one due to PSARP performed elsewhere. Thirty-eight male neonates were thus included in the study (Table 1). Median follow-up time post-PSARP was 8.2 (0.3–15.7) years. No remnant of any of the fistulas was diagnosed during follow-up, while one patient underwent re-operation for anal stenosis and another patient for mucosal prolapse.

**Table 1:** Boys born with ARM reconstructed through PSARP and a divided colostomy

	**Recto-urinary tract fistula**^**b**^ *n* = 31	**No fistula** *n* = 7	***p*-value**
**Prematurity**^**a**^	9 (29)	4 (57)	0.20^e^
**Birth weight** (g)	3020 (1700–4280)	3100 (2450–3895)	0.73^f^
**Small for gestational age**	1 (3)	0	1^e^
**Concomitant malformations**			
Total (at least one)	27 (87)	4 (57)	0.10^e^
Vertebral	19 (61)	1 (14)	*0.04*^e^
Sacral or coccygeal	17 (55)	0	*0.01*^e^
Tethered spinal cord	9 (29)	0	0.16^e^
Caudal regression	3 (10)	0	1^e^
Urinary tract	11 (35)	0	0.08^e^
Genital	5 (16)	0	0.56^e^ 0.56^e^ 0.56^e^
Gastro-intestinal tract	5 (16)	0	
Limb	5 (16)	0	
Cardiac	4 (13)	2 (29)	0.30^e^
Cranio-facial	2 (6)	1 (14)	0.47^e^
**VACTERL association**	12 (39)	0	0.07^e^
**Genetic syndromes**			
Total	3 (10)^c^	6 (86)^d^	< *0.01*^e^
Trisomy 21	0	5 (71)	< *0.01*^e^

Values presented as the absolute number and percentage of patients, n (%), and as median (min–max)

*ARM *anorectal malformations, *PSARP *posterior sagittal anorectal plasty

^a^ Gestational week < 38 + 0

^b^ Recto-bulbar fistula *n* = 8, recto-prostatic fistula *n* = 17, and recto-bladder neck fistula *n* = 6

^c^ Di Georges/CATCH 22, OEIS, and suspected syndrome but non-diagnosed

^d^ Beckwith- Wiedermann, and Trisomy 21

^e^ Fisher’s Exact test, two tailed

^f^ Mann–Whitney U-test, two tailed

### Diagnostic accuracy of fistula presence

**Fig. 1:**
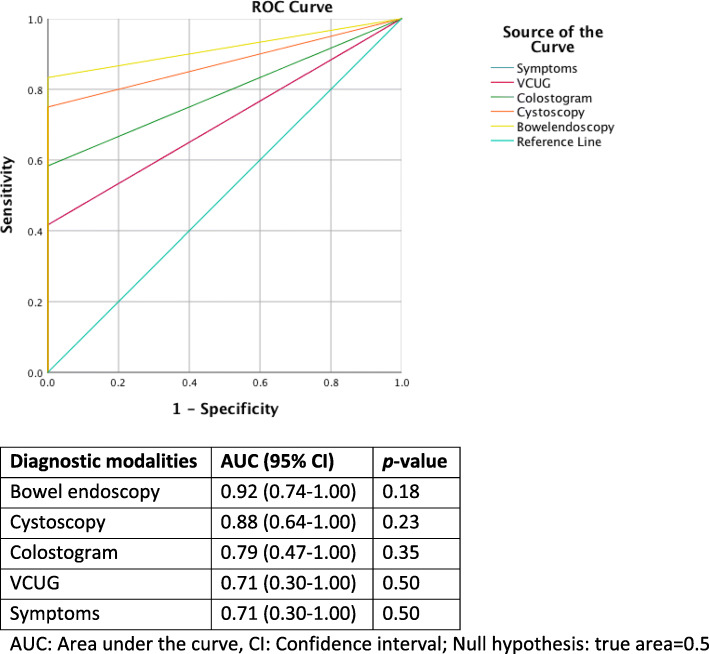
Comparison of diagnostic ability of pre- and peri-operative examinations of fistula presence in boys born with anorectal malformations with final classification during posterior sagittal anorectal plasty

Endoscopy of diverted ostomy had the highest diagnostic accuracy of fistula presence with 22 correctly classified of 24 examined cases (92%). High-pressure colostogram, cystoscopy and VCUG, showed falling diagnostic accuracy (71, 70 and 64% respectively). Symptoms of fistula presence had the lowest diagnostic accuracy; only 21 cases of 38 (55%) were observed correctly (Table 2). Correspondingly, in AUCs calculated from a ROC-curve, endoscopy of diverted bowel showed the highest diagnostic ability of fistula presence, and VCUG and symptoms the lowest (Fig. 1; Comparison of diagnostic ability of pre- and peri-operative examinations of fistula presence in boys born with anorectal malformations with final classification during posterior sagittal anorectal plasty). None of the diagnostic modalities showed any statistically significant superiority.

**Table 2:** Diagnostic accuracy of pre- and peri-operative examinations for fistula presence in boys born with ARM with final classification during PSARP. *n* = numbers, (%) = percent

	**Fistula**^**a**^ *n* = 31	**No fistula** *n* = 7	**Diagnostic accuracy**^**b**^
**Symptoms**	14 (45)	0	21/38 (55)
Stool colored urine	8 (26)	-	
UTI	5 (16)	-	
Urine in colostomy	5 (16)	-	
**VCUG**	29	7	23/36 (64)
*Visible fistula*	16 (55)	0	
**Colostogram**	28	7	25/35 (71)
*Visible fistula*	18 (64)	0	
**Cystoscopy**	19	1	14/20 (70)
*Visible fistula*	13 (68)	0	
**Ostomy endoscopy**	19	5	22/24 (92)
*Visible fistula*	17 (89)	0	
Guide wire used	11 (58)	-	

Values presented as the absolute number and percentage of patients, n (%)

*ARM* anorectal malformations, *PSARP* posterior sagittal anorectal plasty, *UTI* urinary tract infection, *VCUG* voiding cystourethrogram

^a^Recto-bulbar fistula *n* = 8, recto-prostatic fistula *n* = 17, and recto-bladder neck fistula *n* = 6

^b^Diagnostic accuracy (%) = (true positive cases + true negative cases) / all evaluated cases

None of the modalities delivered any false-positive findings. No bowel perforations occurred during high-pressure colostograms. No complications occurred during distal ostomy endoscopy including during fistula catheterization with a guide wire.

### Diagnostic accuracy of fistula level determination

Cystoscopy had the highest diagnostic accuracy of fistula level determination, correctly classifying 14 of 20 examined cases (70%), closely followed by high-pressure colostogram where 23 of 35 examined cases (66%) were classified correctly. VCUG had the lowest diagnostic accuracy of fistula level determination; 21 of 36 examined cases (58%) were classified correctly (Table 3).

**Table 3:** Diagnostic accuracy of pre- and peri-operative examinations of fistula level determination in boys born with ARM with final classification during PSARP. *n* = numbers, (%) = percent

	**Fistulae** *n* = 31			**No fistula** *n* = 7	**Diagnostic accuracy**^**a**^
	**Recto-bulbar** *n* = 8	**Recto-prostatic** *n* = 17	**Bladder neck** *n* = 6		
**VCUG**	8	15	6	7	21/36 (58)
*Correct fistula level determination*	5 (63)	8 (53)	1 (17)	7 (100)	
**Colostogram**	8	15	5	7	23/35 (66)
*Correct fistula level determination*	5 (63)	8 (53)	3 (60)	7 (100)	
**Cystoscopy**	4	12	3	1	14/20 (70)
*Correct fistula level determination*	2 (50)	9 (75)	2 (67)	1 (100)	

Values presented as the absolute number and percentage of patients, n (%)

*ARM* anorectal malformations, *PSARP* posterior sagittal anorectal plasty, *VCUG* voiding cystourethrogram

^a^Diagnostic accuracy (%) = (true positive cases + true negative cases) / all evaluated cases

## Discussion

In this study, ostomy endoscopy and high-pressure colostogram had the highest diagnostic accuracy for fistula presence in ARM, while cystoscopy and high-pressure colostogram had the highest accuracy for fistula level determination. Correct pre-operative ARM-typing only reached a maximum of 60–70% and no modality was proven statistically superior to any others.

Even though this study revealed ostomy endoscopy to be reliable with 92% diagnostic accuracy of fistula presence, pre-operative information solely regarding fistula presence is not enough for the responsible pediatric surgeon. In planning a safe PSARP, an accurate pre-operative predictive anatomic model of each malformation is desirable, preferably visualizing anatomic details and possible potential obstacles [3, 9, 20, 23].

This study from one ARM-center, with quite low patient volumes until 2018 when it was appointed as a national center, revealed fairly poor individual diagnostic accuracy of all analyzed modalities of fistula level determination.

According to the literature, high-pressure colostogram should be the gold standard radiologic method to determine both fistula presence and level in ARM [13, 15]. Our results confirm colostogram to be a robust modality for determining fistula presence but weaker than expected for fistula level determination. To improve pre-operative fistula diagnostics, it is important to ensure that high-pressure colostogram is performed according to the literature-described correct method [14]. High-pressure colostogram has apparent limitations as a diagnostic method due to its operator-dependent outcome which is compromised in low-volume centers; in addition it is a source of radiation and there is the risk of bowel perforation [24, 25].

VCUG has been highlighted as a safe method with high accuracy for fistula level determination, although it is also subjected to method limitations including operator-dependent outcome [14, 15, 26, 27]. VCUG is easier to perform in younger immobile infants compared to older children. To optimize fistula visualization, it is imperative to use VUCG contrast catheters with only one end-opening and not several side-openings. Catheterization may be difficult due to urethral anatomic alterations in ARM with recto-urethral fistulas. According to the clinical experience of pediatric radiologists in our department, a synergetic effect when performing VCUG and colostogram simultaneously may improve fistula diagnostic accuracy. Such simultaneous examinations of colostogram and VCUG were not implemented fully in our department until a couple of years ago, and corresponding data were therefore not analyzed in the present study due to there only being a few cases.

Recent reports of ultrasound- and MRI-examinations have revealed advantages in pre-operative diagnostics in ARM by reducing radiation, improving accuracy of fistula level determination and enabling simultaneous diagnostics of concomitant malformations of the spine and sacrum, spinal cord, genitalia and pelvic floor muscle complex [15, 17–21]. MRI method limitations are need for anesthesia and current limited imaging resolution in infants, and ultrasound is operator dependent. To enable accurate visualization and subsequent pre-operative anatomic models of fistulas and pelvic floor anatomy, method development, assessment and proved safety in children of high-Tesla MRI and validation of high-frequency 3D/4D ultrasound are needed. Printed 3D-anatomic models might contribute to better pre-operative planning and understanding of the complex malformations.

Strengths of this study include a broad inclusion population from a national ARM-center with a standardized program of pre-operative ARM-diagnostics and long-term follow-up involving only a handful of radiologists and pediatric surgeons. Limitations are the retrospective study design with no secondary review of X-ray reports and only a few included patients meaning that it was not possible to show statistically proven differences in diagnostic ability.

## Conclusions

This study reveals that distal ostomy endoscopy has the highest diagnostic accuracy for fistula presence and cystoscopy and high-pressure colostogram has the highest diagnostic accuracy for fistula level determination. Correct pre-operative ARM-typing only reached a maximum of 60–70% and no modality was proven statistically superior to any others.

## Data Availability

The datasets used during the current study are available from the corresponding author on reasonable request.

## Abbreviations

ARM: Anorectal malformations
PSARP: Posterior sagittal anorectal plasty
UTI: Urinary tract infection
VCUG: Voiding cystourethrogram
MRI: Magnetic resonance imaging

## References

[1] Levitt MA, Peña A (2007). Anorectal malformations. Orphanet J Rare Dis.

[2] van der Steeg HJJ, Schmiedeke E, Bagolan P, Broens P, Demirogullari B, Garcia-Vazquez A (2015). European consensus meeting of ARM-Net members concerning diagnosis and early management of newborns with anorectal malformations. Tech Coloproctol.

[3] Bischoff A, Levitt MA, Peña A (2013). Update on the management of anorectal malformations. Pediatr Surg Int.

[4] Holschneider A, Hutson J, Peña A, Bekhit E, Chatterjee S, Coran A (2005). Preliminary report on the international conference for the development of standards for the treatment of anorectal malformations. J Pediatr Surg.

[5] Kyrklund K, Pakarinen MP, Rintala RJ (2017). Long-term bowel function, quality of life and sexual function in patients with anorectal malformations treated during the PSARP era. Semin Pediatr Surg.

[6] Danielson J, Karlbom U, Graf W, Wester T (2017). Outcome in adults with anorectal malformations in relation to modern classification — which patients do we need to follow beyond childhood?. J Pediatr Surg.

[7] Stenström P, Clementson Kockum C, Katsianikou Benér D, Ivarsson C, Arnbjörnsson E (2014). Adolescents with anorectal malformation: physical outcome, sexual health and quality of life. Int J Adolesc Med Health.

[8] Peña A, Devries PA (1982). Posterior sagittal anorectoplasty: important technical considerations and new applications. J Pediatr Surg.

[9] Bischoff A, Bealer J, Wilcox DT, Peña A (2019). Error traps and culture of safety in anorectal malformations. Semin Pediatr Surg.

[10] Halleran DR, Ahmad H, Bates DG, Vilanova-Sanchez A, Wood RJ, Levitt MA (2019). A call to ARMs: accurate identification of the anatomy of the rectourethral fistula in anorectal malformations. J Pediatr Surg.

[11] Bischoff A, Peña A, Levitt MA (2013). Laparoscopic-assisted PSARP - the advantages of combining both techniques for the treatment of anorectal malformations with recto-bladderneck or high prostatic fistulas. J Pediatr Surg.

[12] Hong AR, Acuña MF, Peña A, Chaves L, Rodriguez G (2002). Urologic injuries associated with repair of anorectal malformations in male patients. J Pediatr Surg.

[13] Kraus SJ, Levitt MA, Peña A (2018). Augmented-pressure distal colostogram: the most important diagnostic tool for planning definitive surgical repair of anorectal malformations in boys. Pediatr Radiol.

[14] Abdalla WMA, De La Torre L (2017). The high pressure distal colostogram in anorectal malformations: technique and pitfalls. J Pediatr Surg.

[15] Westgarth-Taylor C, Westgarth-Taylor T, Wood R, Levitt M (2015). Imaging in anorectal malformations: what does the surgeon need to know?. South Afr J Radiol.

[16] Stenström P, Anderberg M, Kockum C, Arnbjornsson E (2013). Endoscopically placed rectourethral guidewire facilitates the reconstruction of anus in children with anorectal malformations: a case report. Eur J Pediatr Surg.

[17] Thomeer MG, Devos A, Lequin M, De Graaf N, Meeussen CJHM, Meradji M (2015). High resolution MRI for preoperative work-up of neonates with an anorectal malformation: a direct comparison with distal pressure colostography/fistulography. Eur Radiol.

[18] Hosokawa T, Yamada Y, Tanami Y, Sato Y, Ishimaru T, Tanaka Y (2019). Comparison of diagnostic accuracy for fistulae at ultrasound and voiding cystourethrogram in neonates with anorectal malformation. Pediatr Radiol.

[19] Hosokawa T, Yamada Y, Hsokawa M, Kikuchi S, Ohira K, Tanami Y (2018). Ultrasound imaging of the anorectal malformation during the neonatal period: a comprehensive review. Jpn J Radiol.

[20] Madhusmita, Ghasi RG, Mittal MK, Bagga D (2018). Anorectal malformations: role of MRI in preoperative evaluation Indian. J Radiol Imaging.

[21] Podberesky DJ, Towbin AJ, Eltomey MA, Levitt MA (2013). Magnetic resonance imaging of anorectal malformations. Magn Reson Imaging Clin N Am.

[22] Fernbach SK, Feinstein KA, Schmidt MB (2000). Pediatric voiding cystourethrography: a pictorial guide. Radiographics.

[23] Bischoff A, Bealer J, Peña A (2017). Controversies in anorectal malformations. Lancet Child Adolesc Heal.

[24] Brisighelli G, Lorentz L, Pillay T, Westgarth-Taylor CJ (2020). Rectal perforation following high-pressure distal colostogram. Eur J Pediatr Surg Reports.

[25] Midrio P, van Rooij IALM, Brisighelli G, Garcia A, Fanjul M, Broens P (2020). Inter- and intraobserver variation in the assessment of preoperative colostograms in male anorectal malformations: an ARM-net consortium survey. Front Pediatr.

[26] Malhotra NR, Green JR, Rigsby CK, Holl JL, Cheng EY, Johnson EK (2017). Urinary tract infection after retrograde urethrogram in children: a multicenter study. J Pediatr Urol.

[27] Alamo L, Meyrat BJ, Meuwly JY, Meuli RA, Gudinchet F (2013). Anorectal malformations: finding the pathway out of the labyrinth. Radiographics.

